# Qinbaohong Zhike Oral Liquid Attenuates LPS-Induced Acute Lung Injury in Immature Rats by Inhibiting OLFM4

**DOI:** 10.1155/2022/7272371

**Published:** 2022-08-16

**Authors:** Fangbo Zhang, Yu Li, Yujie Xi, Yi Zhang, Lifang Wang, He Xu, Jixiang Tian, FeiFei Guo, Hongjun Yang

**Affiliations:** ^1^Institute of Chinese Materia Medica, China Academy of Chinese Medical Sciences, Beijing, China; ^2^Tianjin University of Traditional Chinese Medicine, Tianjin, China; ^3^Experimental Research Center, China Academy of Chinese Medical Sciences, Beijing, China

## Abstract

Acute respiratory infections (ARIs) are a common public safety threat with high morbidity and mortality in pediatric patients worldwide. Qinbaohong Zhike oral liquid (QBH), a marketed traditional Chinese medicine product, has been widely used to cure respiratory diseases. QBH is reported to have antitussive, expectorant, and antiasthmatic properties. However, its treatment effect against ARIs is not elucidated. This study aimed to explore the therapeutic efficacy of QBH in the treatment of ARIs-induced pneumonia. Network pharmacology was used to predict the possible targets of QBH against ARIs. Next, the tracheal lipopolysaccharide (LPS-)-induced acute lung injury (ALI) immature rat model was constructed to evaluate the therapeutic effect of QBH. Tandem mass tag (TMT-)-based quantitative proteomics was then used to screen the in-depth disease targets of QBH. QBH exerted a protective effect against LPS-induced ALI by inhibiting pulmonary pathological damage. QBH also reduced the levels of interleukin (IL)-6, tumor necrosis factor (TNF)-*α*, interferon (IFN)-*γ*, and granulocyte macrophage colony-stimulating factor (GM-CSF) in the serum and IL-1*β*, IL-6, IL-8, TNF-*α*, IFN-*γ*, and GM-CSF in the lung tissue. Based on proteomic data, olfactomedin 4 (OLFM4) related to immunity and inflammation was selected as a potential target. Western blot analysis further confirmed the moderating effect of QBH downregulation on OLFM4 in the lung tissue. Our findings demonstrated that QBH alleviated lung tissue damage and inflammatory reaction via inhibiting OLFM4 expression in LPS-challenged immature rats. Our research indicates that QBH may have therapeutic potential for treating ARIs-related ALI in pediatric patients, which also serves as a candidate target for drug therapy of ALI by intervening OLFM-related signaling pathways.

## 1. Introduction

Acute respiratory infections (ARIs) such as bronchiolitis and pneumonia in pediatric patients remain a major public health problem worldwide [1]. ARIs-induced pneumonia is one of the major causes of morbidity and mortality [2, 3]. The incidence rate of ARIs varies among children, and the severity of ARIs ranges from mild to severe [4]. However, the underlying causes for these variations are not precisely known [5]. Respiratory viruses such as respiratory syncytial virus, rhinovirus, and influenza virus are the main ARIs-causing pathogens [6]. The bacteria including normal commensal bacteria existing in the respiratory tract can also cause infections. Nasal bacteria such as M. catarrhalis and Streptococcus pneumoniae are mostly asymptomatic, but sometimes lead to pulmonary infections even sepsis via mucosal transfer or systemic infection [7]. Severe viral or bacterial infection can result in acute lung injury (ALI), which is a critical pathological event that may develop into acute respiratory failure.

Current clinical treatments are available to relieve symptoms and shorten illness duration, albeit these therapies have limited efficacy as well as side effects. For example, although vaccination helps prevent infections, they are insufficient during outbreaks of new infectious with pandemic potential [8]. Furthermore, vaccines may be less effective or have serious adverse effects in certain patients [9]. Antibiotics are the primary therapy to treat a bacterial infection, although their efficacy is rapidly declining because of antibiotic resistance incurred by the long-term application and misuse [10, 11]. Antiviral medicines may have more adverse effects, such as headache, nausea, vomiting, and gastrointestinal problems [12, 13]. Therefore, detailed research focusing on the identification of new drug targets and the development of novel therapeutic agents is urgently warranted.

Traditional Chinese medicine (TCM-)-based herbal therapies are being used to treat infectious diseases for almost two thousand years in China [14]. These therapies show fewer side effects and possess a milder cure process against infections. Qinbaohong Zhike oral liquid (QBH) contains three TCM herbs: *Rhododendron dauricum* L. (Man-Shan-Hong), *Syringa reticulata* (Blume) H. Hara (Bao-Ma-Zi-Pi), and *Scutellaria baicalensis* Georgi (Huang-Qin). QBH has been clinically used to treat lung phlegm heat syndrome in patients with acute or chronic bronchitis. QBH has antitussive, expectorant, and antiasthmatic properties [15], but the therapeutic efficacy against ARIs is not completely elucidated. Lipopolysaccharide (LPS) is a major component of cell wall of gram-negative bacteria. Intratracheal administration of LPS leads to acute pulmonary inflammation, which is characterized by lung edema, destruction of epithelial barrier integrity, recruitment of neutrophils, and release of inflammatory factors [16]. Presently, traditional Chinese medicines (TCMs) for treating ARIs in child patients are limited. Clinical applications have shown that QBH has definite therapeutic properties. Meanwhile, the formula of QBH is simple, only contains three herbs, which may bring fewer side effects and be more suitable for pediatric patients.

In the current study, we used network pharmacology to predict the possible pharmacological mechanism of QBH in the treatment of ARIs, which could provide the theoretical foundation [17]. Then, we established an ALI immature rat model via intratracheal administration of LPS to analyze the therapeutic effect of QBH. We further screened olfactomedin 4 (OLFM4) by quantitative proteomics to determine its eligibility as a potential target for ALI treatment. The human olfactomedins (OLFMs) family is reported to play important physiological functions mainly related to immunity and inflammation [18]. Specifically, OLFM4 has been confirmed to be associated with disease severity in children with viral lower respiratory tract infections [19]. In the light of these findings, our study may provide scientific evidence for the mechanistic assessment and clinical application of QBH in treating ALI in children, and our results characterized the OLFM4 expression in the lung tissues of the immature rats with LPS-induced ALI to illustrate the role of OLFM4 in ARIs.

## 2. Materials and Methods

### 2.1. Network Pharmacology Analysis of QBH in the Treatment of ARIs

The information about the chemical components of QBH was collected from the Traditional Chinese Medicine Systems Pharmacology (TCMSP) database (https://tcmspw.com/tcmsp.php). Documentary records were also used to supplement the TCMSP database result [20–22]. The compounds with drug likeness (DL) ≥0.18 and oral bioavailability (OB) ≥30% were selected as active compounds. The potential molecular targets for respiratory tract infections were acquired from Human Phenotype Ontology (https://hpo.jax.org/app/) and DisGeNET databases (http://www.disgenet.org), due to ARIs not on the list of search terms in these two databases.

We merged drug targets of QBH and disease targets of respiratory tract infections. These targets were loaded into the STRING database (http://string-db.org) to acquire the core protein-protein interaction (PPI) network. The PPI network was built by the Cytoscape software (version 3.7.2, Boston, MA, USA). To determine the potential biological functions of QBH, we used the Metascape database (http://metascape.org/gp/index) to perform Gene Ontology (GO) and Kyoto Encyclopedia of Genes and Genomes (KEGG) pathway enrichment analysis. The GO enrichment analysis included three terms: biological process (BP), cellular component (CC), and molecular function (MF).

### 2.2. Experimental Animals and Drugs

Male immature Sprague-Dawley rats aged 18 to 21 days were obtained from Beijing HFK Bioscience Co. Ltd. (Beijing, China) with the license number SCXK (Jing) 2016-0004. The rats were raised in the SPF animal room of the Institute of Chinese Materia Medica, China Academy of Chinese Medical Sciences, Beijing, China. All animal procedures were performed according to the local ethical guidelines for animal experiments of the Animal Research Committee of the China Academy of Chinese Medical Sciences (Approval No. 2020B029).

QBH consists of three TCMs: Huang-Qin, Man-Shan-Hong, and Bao-Ma-Zi-Pi (weight ratio10: 21: 21). The herbal extract was prepared by Heilongjiang Bifu Jinbeiyao Biopharmaceutical Co. Ltd., China. The extract was dissolved in distilled water. The clinical oral dose of QBH was 0.5 mL/kg/day (60 kg, 10 mL/time, t.i.d.), which was equivalent to the gavage dose of 3.125 mL/kg/day for the rats (200 g) [15]. Thus, we set three gavage doses involving 1.5, 3.0, and 6.0 mL/kg/day. Dexamethasone tablets (DXMS, 0.75 mg/tablet) were purchased from Guangdong Huanan Pharmaceutical Group Co. Ltd., China.

### 2.3. Establishment of an Immature Rat ALI Model

The immature rat model of ALI was established as described [23]. Briefly, the immature rats were anesthetized with 1% pentobarbital sodium (25 mg/kg, Sigma, MO, USA) via intraperitoneal injection, and then administered with a tracheal injection of 60 *μ*L LPS (1 *μ*g/*μ*L, in 0.9% sterile saline, from Escherichia coli 055: B5, cat. No. L2880, Sigma, MO, USA) through an endotracheal tube (mouse 20G type, Zhongyanboji, Beijing, China). All rats were randomly assigned to six groups (*n* = 12 each): control group (control), ALI model group (ALI), QBH low-dose group (ALI + QBH 1.5 mL/kg), QBH medium-dose group (ALI + QBH 3.0 mL/kg), QBH high-dose group (ALI + QBH 6.0 mL/kg), and dexamethasone-treated group (ALI + DXMS 0.42 mg/kg) [24, 25]. Control group rats were given 60 *μ*L sterile saline through the trachea. At 6 h after anesthesia, QBH or DXMS group rats were administered intragastrically with QBH or DXMS for 3 consecutive days. Control and ALI group rats were given distilled water by gavage.

### 2.4. Lung Wet/Dry (W/D) Weight Ratio Measurement

On the 4th day, the immature rats were killed, and the lung tissue of each rat was removed. The lobe of the left lung was isolated and weighed quickly to prevent fluid loss (wet weight). Next, it was dried in an oven (Hengfeng, Hubei, China) at 80°C for 24 h and then measured (dry weight). The calculated W/D weight ratio reflected the extent of pulmonary edema.

### 2.5. Pulmonary Pathological Assessment

The middle lobe of the right lung was immediately removed, fixed in 4% paraformaldehyde solution (Solarbio, Beijing, China), embedded in the paraffin wax, sliced in 5-*μ*m-thick sections, and stained with hematoxylin and eosin (HE). The tissue slides were observed under a light microscope (Olympus, Tokyo, Japan) for conventional morphological evaluation. The histological score was calculated as follows: (i) alveolar congestion; (ii) alveolar hemorrhage; (iii) interstitial edema; (iv) neutrophil infiltration; and (v) the thickness of the alveolar wall. Each category was scored as 0 for no injury, 1 for slight injury, 2 for moderate injury, and 3 for severe injury [26].

### 2.6. Inflammatory Cytokines Detection

Peripheral blood from the retro-orbital venous plexus and lung tissue from the middle lobe of the right lung was collected for detecting the inflammatory cytokines. The blood sample was centrifuged at 1,500*g* for 10 min, and the serum in the upper layer was gained. The tissue sample was lysed using ice-cold RIPA lysis buffer and protease inhibitors (Solarbio, Beijing, China). Subsequently, the sample was crushed by the ultrasonic wave (Xinzhi, Ningbo, China) and then centrifuged at 13,000*g* for 15 min (4°C). The concentration of each tissue sample was determined by a BCA protein assay kit (Pierce, CA, USA).

Luminex technology-based multiplex system (BioPlex, BioRad, CA, USA) combined with a multiplex immunoassay kit (BioPlex Pro™ Rat Cytokine Assay, BioRad, CA, USA) was used to determine the levels of the serum and tissue samples [27]. A total of 23 inflammatory cytokines were determined as follows: interleukin (IL)-1*α*, IL-1*β*, IL-2, IL-4, IL-5, IL-6, IL-7, IL-8 (GRO/KC), IL-10, IL-12 (p70), IL-13, IL-17A, IL-18, granulocyte-macrophage colony-stimulating factor (GM-CSF), macrophage colony-stimulating factor (M-CSF), granulocyte colony-stimulating factor (G-CSF), monocyte chemoattractant protein (MCP)-1, macrophage inflammatory protein (MIP)-1*α*, MIP-3*α*, regulated upon activation, normal T cell expressed and presumably secreted (RANTES), vascular endothelial growth factor (VEGF), interferon (IFN)-*γ* and tumor necrosis factor (TNF)-*α*.

### 2.7. Tandem Mass Tag (TMT-)-Based Quantitative Proteomic Analysis

15 biological replicates (*n* = 5/group) were examined from the control group, ALI group, and QBH 6.0 mL/kg treated group. A protein sample from the middle lobe of the right lung tissue was extracted by lysis solution (2% SDS and 7 M urea) mixed with a protease inhibitors cocktail (Sigma, MO, USA) and centrifuged at 13,000*g* for 15 min at 4°C. The supernatant was collected, and the concentration of protein sample was measured by the BCA protein assay kit (Pierce, CA, USA). In brief, the sample was precipitated with acetone-TCA and digested by trypsin to generate proteolytic peptides. The peptides were labeled with 16-plex TMT reagents (Thermo Fisher, MA, USA) according to the manufacturer's instructions. The TMT-labeled samples were separated by the Ultimate 3000 HPLC system (Thermo Fisher, MA, USA). The peptide samples were isolated, centrifuged and dried in a vacuum. The dried samples were dissolved in 0.1% formic acid solution, and 2 *μ*g of the prepared sample was analyzed by an Easy-nLC1000 system (Thermo Fisher, MA, USA). The DAVID database (https://david.ncifcrf.gov/) was used for functional enrichment analysis including GO and KEGG, and the differentially expressed proteins (DEPs) between the groups were analyzed. A *p* value <0.05 with fold change ≥1.2 (upregulated) or ≤0.83 (downregulated) was considered statistically significant.

### 2.8. Western Blot

The middle lobe of the right lung tissue was lysed with RIPA lysis buffer and protease inhibitors (Solarbio, Beijing, China). Protein concentrations were measured by the BCA protein assay kit (Pierce, CA, USA). The protein sample (20 *μ*g) was separated by 10% SDS-PAGE and transferred to PVDF membranes (Millipore, MA, USA). After blocking with 5% non-fat milk at 4°C overnight, the membrane was incubated with primary antibody olfactomedin 4 (cat. No. bs-6558R, 1 : 1,000, Bioss, Beijing, China) overnight at 4°C. *β*-actin (cat. No. 3700, 1 : 1,000, Cell Signaling Technology, MA, USA) was used as an internal control. The membrane was then incubated with a secondary antibody for 4 h at room temperature, and the intensity was measured by an enhanced chemiluminescence agent (Millipore, MA, USA).

### 2.9. Statistical Analysis

Statistical analysis was performed by SPSS 20.0 software. The data were expressed as mean ± standard deviation (SD). Student's *t*-test and one-way analysis of variance (ANOVA) were used for comparisons. A *p* value < 0.05 was regarded as statistically significant.

## 3. Results

### 3.1. Protein-Protein Interaction Network Construction and Functional Enrichment Analysis of QBH against ARIs

**Figure 1:**
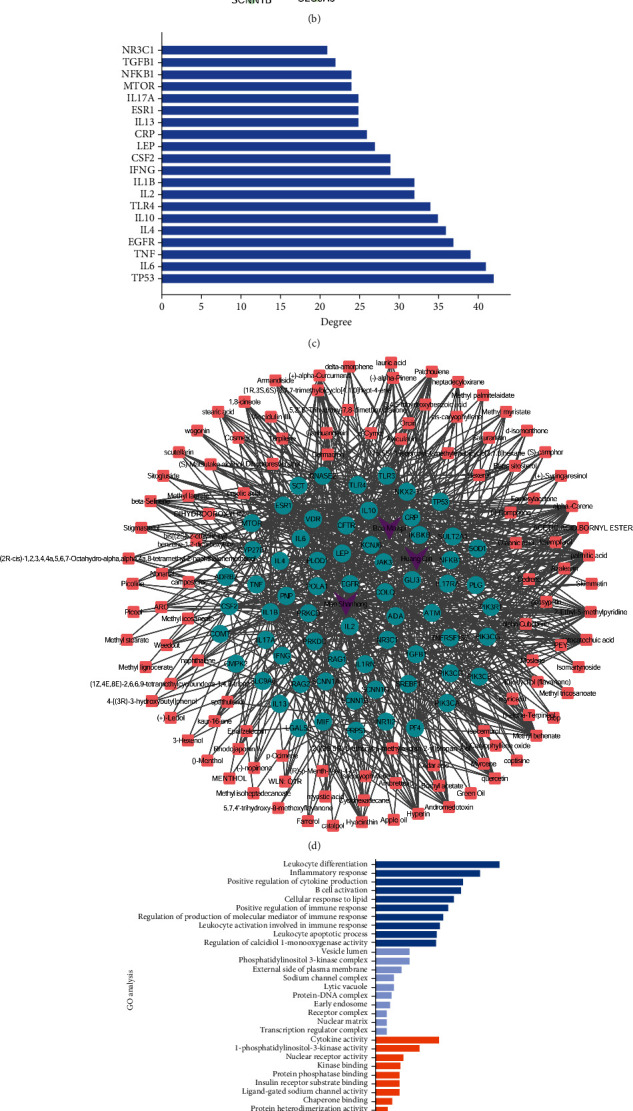
Network pharmacology analysis of QBH in the treatment of ARIs. (a) Venn diagram showing the identified number of 65 shared targets by QBH and ARIs. (b) The PPI network constructed by 65 overlapping genes between QBH targets and ARIs targets. The nodes represented potential genes, and the edges represented protein-protein connections. (c) Horizontal stack plot showing the top 20 target genes in PPI network. (d) A drug-component-target interaction network was constructed. Each herb of QBH, active compounds of QBH, and target genes was represented by the purple, pink, and green, respectively. (e) Functional GO analysis showing the top 10 significant enrichment terms in BP, CC, and MF, respectively. (f) Functional KEGG analysis showing the top 10 enrichment signaling pathways.

In all, 324 ingredients of QBH were identified after removing the duplicated data, including 154 in Man-Shan-Hong, 41 in Bao-Ma-Zi-Pi, and 143 in Huang-Qin, and then, 1,245 potential targets of QBH were collected. Meanwhile, we obtained 358 target genes related to ARIs. There were 65 overlapping genes between QBH targets and ARIs targets (Figure 1(a)). To determine the interactions of overlapping genes, we uploaded the information of these genes in the STRING database and constructed a PPI network (Figure 1(b)). Figure 1(b) shows that 8 inflammatory cytokines such as IL-1*β*, IL-2, IL-4, IL-6, IL-10, IL-13, IL-17*α*, and TNF-*α* were centrally located at the core position of the PPI network. Moreover, the horizontal stack plot displayed the above 8 inflammatory cytokines were also enriched in the top 20 of the PPI network (Figure 1(c)). These results demonstrated that these molecular targets may be highly related to the pathogenesis of ARIs and the treatment of QBH.

To further figure out the possible therapeutic targets, a drug-component-target interaction network was then developed. The network included 178 nodes and 700 edges, indicating the characteristics of multiple target effects correlated with QBH in the treatment of ARIs (Figure 1(d)). Functional GO and KEGG enrichment analyses were performed to elucidate the mechanism of QBH involved in the treatment of ARIs. GO enrichment analysis revealed that BP component was mainly associated with inflammatory reaction and immune response such as leukocyte differentiation, inflammatory response, positive regulation of cytokine production, B cell activation, cellular response to lipid, positive regulation of immune response, regulation of production of molecular mediator of immune response, leukocyte activation involved in immune response, leukocyte apoptotic process, and regulation of calcidiol 1-monooxygenase activity. The CC results suggested that the target genes were mainly located in the vesicle lumen, external side of the plasma membrane, and nuclear matrix. The MF results showed that the majority of targets were associated with the activities of cytokines, 1-phosphatidylinositol-3-kinase, and nuclear receptors (Figure 1(e)). KEGG enrichment analysis indicated that these targets were mainly involved in cytokine-cytokine receptor interaction, Th17 cell differentiation, JAK/STAT, T cell receptor, Toll-like receptor, PI3K/Akt, TNF, HIF-1, AMPK, and chemokine signaling pathways (Figure 1(f)).

### 3.2. QBH Alleviated Lung Tissue Injury Induced by Intratracheal LPS

**Figure 2:**
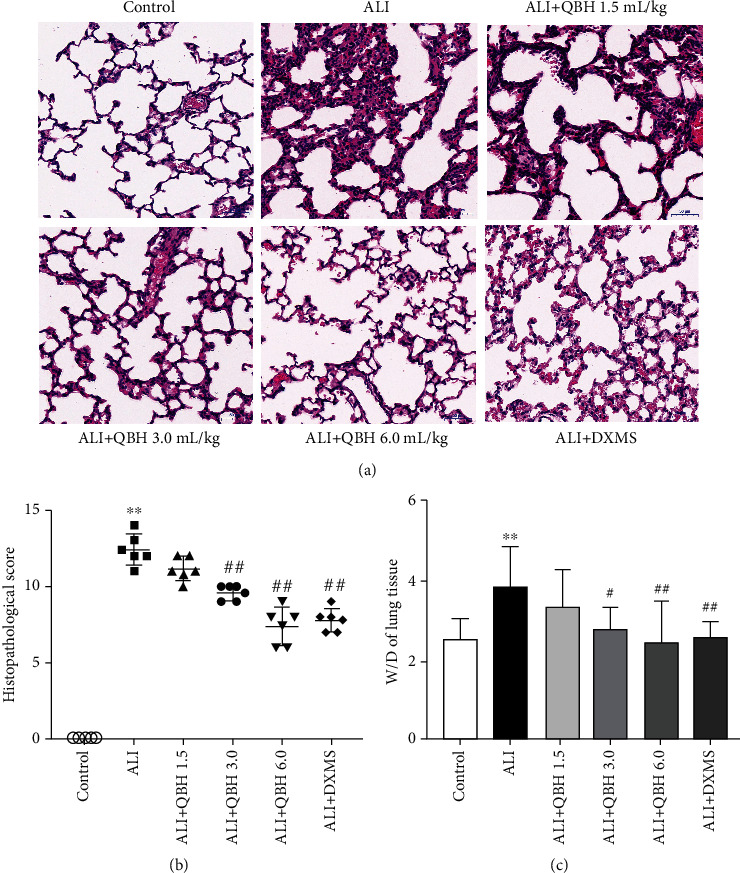
Protective effect of QBH on pulmonary pathological changes of ALI immature rats. (a) Pathological features of lung tissue detected by HE staining (scale bar 50 *μ*m, magnification 200×). (b) Histological score of lung tissue (*n* = 6/group). (c) Lung wet/dry (W/D) weight ratio (*n* = 10/group). Data are presented as mean ± SD. ^∗∗^*p* < 0.01 vs. control group; #*p* < 0.05, and ##*p* < 0.01 vs. ALI group.

Intratracheal administration of LPS in immature rats led to expected pathological changes including pulmonary hyperemia, edema, thickened alveolar walls, and inflammatory cell infiltration. Moreover, the severity of lung tissue injury was alleviated in the QBH 3.0 and 6.0 groups (Figure 2(a)). These histopathological changes also brought the increases in lung injury score (12.40 ± 1.14) and lung W/D weight ratio (3.82 ± 1.04) in the ALI group (Figure 2(b)). Compared with the ALI group rats, rats treated with QBH 3.0 mL/kg (9.60 ± 0.54) and 6.0 mL/kg (7.40 ± 1.34) showed remarkable declines in the lung injury score. Besides, QBH-treated groups (3.0 mL/kg, 2.80 ± 0.51; 6.0 mL/kg, 2.46 ± 1.02) had reduced lung W/D weight ratios compared with the ALI group (Figure 2(c)). High-dose QBH group had the highest protective effect among the three QBH-treated groups.

### 3.3. QBH Inhibited the Levels of Inflammatory Factors in Blood Serum and Lung Tissue

**Figure 3:**
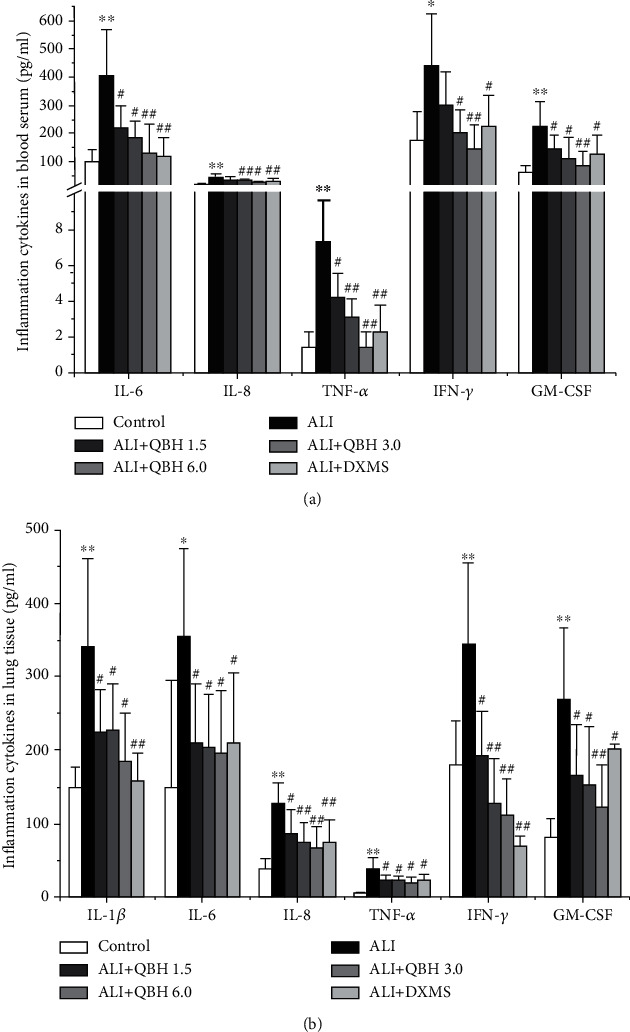
QBH inhibited the levels of inflammatory cytokines in blood serum (a, *n* = 6/group) and lung tissue (b, *n* = 7/group) induced by intratracheal LPS. Data are presented as mean ± SD. ^∗^*p* < 0.05, and ^∗∗^*p* < 0.01 vs. control group; #*p* < 0.05 and ##*p* < 0.01 vs. ALI group.

To validate the network pharmacology result, we determined the levels of 23 inflammatory cytokines in the blood serum and lung tissue. Compared with the control group, the ALI group had increased levels of IL-6, IL-8, TNF-*α*, IFN-*γ*, and GM-CSF in the blood serum, and IL-1*β*, IL-6, IL-8, TNF-*α*, IFN-*γ*, and GM-CSF in the lung tissue (Figures 3(a) and 3(b)). Compared with the ALI group, the QBH-treated groups had significantly reduced serum levels of IL-6, TNF-*α*, and GM-CSF. However, the serum level of IL-8 and IFN-*γ* was downregulated only in QBH 3.0 and 6.0 mL/kg groups. Additionally, the levels of IL-1*β*, IL-6, IL-8, TNF-*α*, IFN-*γ*, and GM-CSF in the lung tissue were significantly decreased in all QBH-treated groups compared with the ALI group.

### 3.4. Elucidation of Potential Therapeutic Targets of QBH Using Quantitative Proteomics

To elucidate the probable therapeutic targets of QBH, we quantified 3,385 proteins from the 15 samples. Data showed that 79 and 8 proteins were significantly upregulated and downregulated, respectively, in the ALI group compared with the control group. We identified 91 DEPs including 52 upregulated and 39 downregulated DEPs in the QBH 6.0 mL/kg group compared with the ALI group. There were 10 overlapping DEPs both changed in these two comparisons.

**Figure 4:**
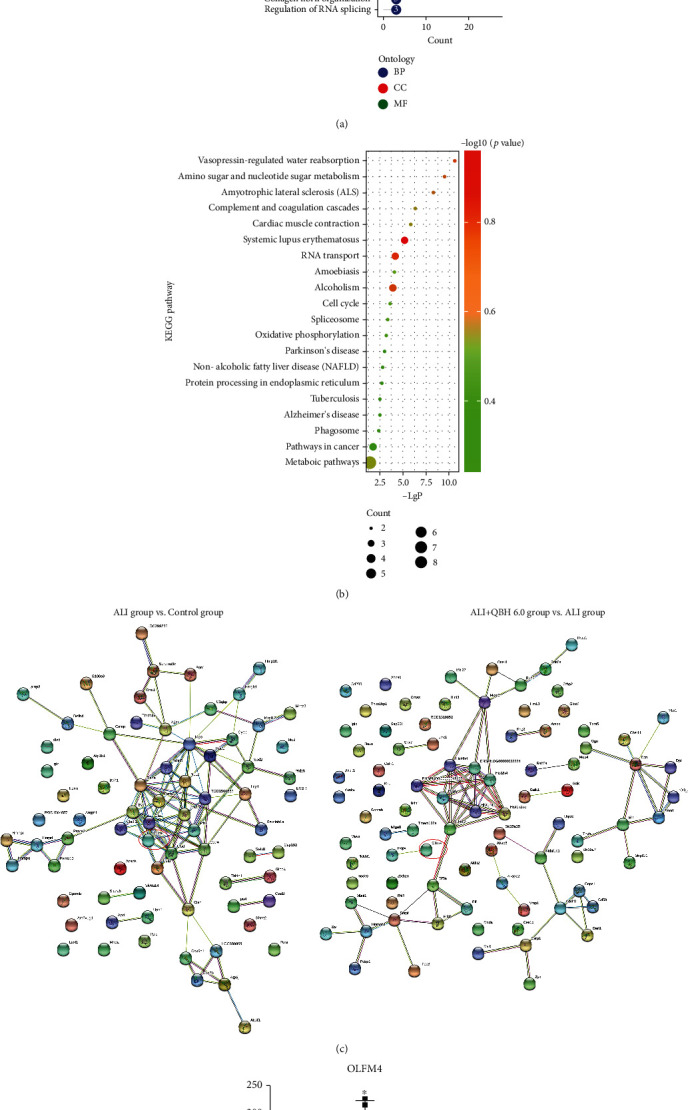
Functional analysis of QBH-regulated DEPs in the lung tissue determined by quantitative proteomics. (a) Functional GO enrichment analysis of the DEPs according to the classification of BP, CC and MF. (b) Functional KEGG enrichment analysis showing the top 20 signaling pathways. (c) The PPI network analysis generated by two different comparisons. (d) Abundances of OLFM4 reported by TMT-based quantitative proteomic result. Data are presented as mean ± SD (*n* = 5/group). ^∗^*p* < 0.05 vs. control group; #*p* < 0.05 vs. ALI group.

GO-enriched categories in BP, CC, and MF were achieved between the ALI group and QBH 6.0 mL/kg group (Figure 4(a)). The BP term of these proteins was mainly involved in the apoptotic process, RNA poly II promoter, protein transport, and RNA splicing. The CC analysis demonstrated that the DEPs were mainly located in extracellular exosomes, cytoplasm, nucleus, and membrane. The MF term revealed that these proteins were primarily connected with protein binding, DNA binding, RNA binding, and ATP binding. KEGG enrichment analysis suggested that signaling pathways of DEPs were related to metabolic pathways, RNA transport, oxidative phosphorylation, and cell cycle (Figure 4(b)).

Two PPI networks were constructed to further explore the potential therapeutic targets underlying the therapeutic mechanism of QBH against ALI (Figure 4(c)). One significant and overlapping protein olfactomedin 4 (OLFM4) was found to be contained in these two networks (Figure 4(d)). According to the items of GO analysis, OLFM4 may be related to the apoptotic process (BP) and protein homooligomerization activity (MF), and it may be located in extracellular space, plasma membrane, and perinuclear region of cytoplasm (CC). Considering the essential role in cellular apoptosis, OLFM4 was selected for further biological validation.

### 3.5. Identification of Protein Target of QBH Based on the Validation of the DEPs

**Figure 5:**
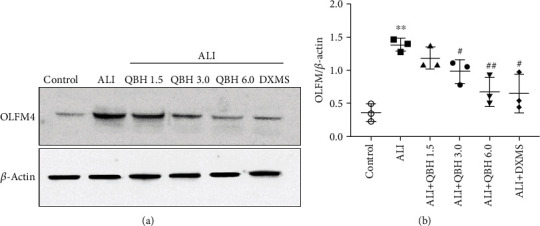
Validation of QBH on the expression of OLFM4 in the lung tissue. (a) Representative images of OLFM4 expression determined by western blot. (b) Relative protein expression level of OLFM4. Data are presented as mean ± SD (*n* = 3/group). ^∗∗^*p* < 0.01 vs. control group; #*p* < 0.05 and ##*p* < 0.01 vs. ALI group.

To validate the differential expression data of proteomic analysis, western blot was performed to detect the expression of OLFM4 in the lung tissue (Figure 5(a)). OLFM4 expression was significantly increased in the ALI group (1.39 ± 0.10) compared with the control group (0.36 ± 0.13), and it was decreased in the QBH-treated groups (3.0 mL/kg, 0.98 ± 0.18; 6.0 mL/kg, 0.67 ± 0.22) compared with the ALI group (Figure 5(b)). The change in the expression of OLFM4 protein was consistent with the result of quantitative proteomics analysis.

## 4. Discussion

ARIs are one of the primary infections in the pediatric population especially in developing countries [28]. Most children may experience three to six ARIs annually. Although these infections are self-limiting, symptoms can be distressing. Emerging evidence strongly suggests that oral homeopathic medicinal products are effective and safe compared with allopathic medicines in the prevention and treatment of ARIs in children [29]. ALI is one of the complications of ARIs, which is difficult to cure and has a poor prognosis. In our research, we established an LPS-induced ALI immature rat model to evaluate the therapeutic effect of QBH. We hoped that the TCM formula QBH may be developed into an effective therapy for treating ARIs-related ALI in child patients.

Lipopolysaccharide is the most common toxin used to stimulate pulmonary inflammation in rodent models of bacterial infection-induced ALI. The resultant innate immune response has a profound effect on the pathogenesis of ALI [30]. Neutrophils and macrophages are the main inflammatory cells and the fundamental source of inflammatory mediators; they are also responsible for multiple immunological processes and tissue injury in ALI [31, 32]. During the acute stage of ALI, the excessive activation of neutrophils and macrophages is one of the most significant factors exacerbating the inflammatory response [33]. The recruited inflammatory cells infiltrate the lung tissue, and release various inflammatory cytokines, which increases the permeability of the alveolar–capillary barrier, eventually resulting in pulmonary dysfunction [34]. The oversecretion of inflammatory cytokines such as interferons (IFNs), tumor necrosis factors (TNFs), interleukins (ILs), and chemokines plays a vital role in the initiation and acceleration of the inflammatory cascade [35].

Past reports suggest that multiple signal transduction pathways, including TLR4/NF-*κ*B, PI3K/Akt, JAK/STAT, and AMPK, are involved in mediating pulmonary inflammation [36]. For example, lipopolysaccharide can activate the TLR4/NF-*κ*B signaling pathway and promote the transcription of downstream cytokines, such as IL-6, TNF-*α*, IL-1, and chemokines [37]. The PI3K/Akt signaling pathway is closely associated with the pulmonary inflammatory reaction by affecting the downstream molecular expression of MAPKs, iNOS, and COX-2 [38]. The abnormal activation of the JAK/STAT pathway is important for persistent inflammation in several pathological conditions involving autoimmunity and infection [39]. The signaling molecule AMPK is the key energy sensor of cellular metabolisms, such as neurodegeneration, inflammation, and oxidative stress [40]. Increasing evidence supports that AMPK acts as a negative modulator of inflammatory responses and plays a protective role in LPS-induced ALI [41]. These research findings signify the significance of the inflammatory response in acute lung diseases, and the suppression of the generation of inflammatory cytokines has been considered a therapeutic measure for the treatment of ALI [42].

In our study, network pharmacology results showed that QBH may probably regulate inflammatory reaction and immune response through the modulation of the critical inflammatory cytokines including IL-1*β*, IL-2, IL-4, IL-6, IL-10, IL-13, IL-17*α*, and TNF-*α*. Moreover, the signaling pathways such as a cytokine-cytokine receptor, T cell receptor, Toll-like receptor, JAK/STAT, PI3K/Akt, and AMPK may be involved and play an important role in the therapeutic mechanism of QBH against ARIs. Animal experiments confirmed that QBH downregulated the levels of IL-6, TNF-*α*, IFN-*γ*, and GM-CSF in the blood serum, and IL-1*β*, IL-6, IL-8, TNF-*α*, IFN-*γ*, and GM-CSF in the lung tissue, and thus protected the lung tissue from LPS-induced injury. The data of IL-1*β*, IL-6, and TNF-*α* were consistent with the network pharmacology result.

To further elucidate the potential therapeutic targets of QBH in the treatment of ALI, we used quantitative proteomics to discover the candidate biomarkers. OLFM4 was selected and then validated by western blot. Western blot result was similar to the result of quantitative proteomic analysis, which indicated high reliability and consistency. The human OLFMs family includes OLFM1, OLFM2, OLFM3, OLFM4, myocilin, gliomedin, latrophilin1, latrophilin2, and latrophilin3 [43]. They are olfactomedin domain-containing glycoproteins known to be the key regulators in a variety of biological functions. Human OLFM4 is mainly expressed in the prostate, bone marrow, and gastrointestinal tracts such as the stomach, small intestine, and colon [44]. It plays an important role in several cellular functions including proliferation, differentiation, apoptosis, and cell adhesion [45]. OLFM4 is confirmed to be the target of the Notch signaling pathway and participates in Notch-mediated differentiation, proliferation, and immune response [46]. OLFM4 is regarded as a marker of asthma, septic shock, and many types of cancers [47–49].

OLFM4 regulated the host defense mechanism against H. pylori infection and negatively regulated the NOD-induced NF-*κ*B signaling pathway via feedback control [18]. OLFM4 deletion enhanced immune response against S. aureus in mice with the chronic granulomatous disease, and OLFM4 may be a critical target for the augmentation of the host defense system against bacterial infection [50]. OLFM4 expression in human neutrophils was upregulated in response to a wide range of bacterial infections, including Gram-positive S. aureus, Gram-negative E. coli and S. enterica infections [51]. OLFM4 expression was increased in gastric biopsies from H. pylori-infected patients [52]. OLFM4 gene expression in the whole blood was upregulated in septic patients with acute respiratory distress syndrome [53]. OLFM4 contributed to H_2_O_2_-induced NADPH oxidase activation and cellular apoptosis in mouse neutrophils [54]. Conversely, the contradictory results of OLFM4 on inflammation have been reported in several studies. For example, OLFM4 expression was downregulated in LPS-induced lung epithelial cells, and it exhibited anti-inflammatory activity through modulating metabolic disorders [55].

Briefly, current evidence demonstrates that OLFM4 is an important regulator of inflammatory and immune responses. In our study, we found that OLFM4 expression was upregulated in the lung tissue of the immature rats with ALI, and QBH significantly inhibited the expression of OLFM4. These results meant that QBH exerted considerable influence on inflammatory and immune responses by altering OLFM4 expression. OLFM4 may be a potential target marker correlated to the pathogenesis of ALI and the therapeutic mechanism of QBH.

## 5. Conclusions

**Figure 6:**
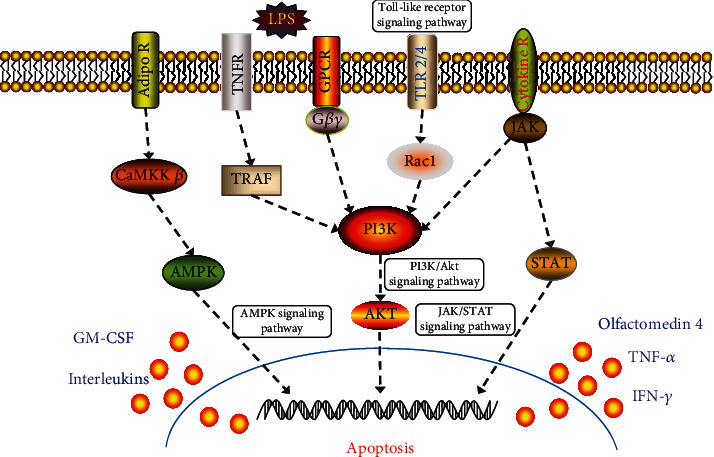
Graphical representation of the main signaling pathways of QBH in the treatment of LPS-induced ALI.

In summary, we used network pharmacology to search for the potential molecular targets of QBH in the treatment of ARIs. An experimental study on immature rats suggested that QBH inhibited lung injury and inflammatory reaction, resulting in ameliorating the severity of ALI induced by intratracheal LPS. Quantitative proteomics and western blot further provided evidence that OLFM4 might be the critical candidate biomarker involved in the therapeutic mechanism of QBH against ALI (Figure 6). Our research provided further insight into the development of therapeutic strategies for treating ALI by regulating OLFM-related signaling pathways. Moreover, our study may offer an experimental foundation for effectively treating ARIs-related ALI in pediatric patients.

## Data Availability

The data that support the findings of this study are available on request from the corresponding author.

## Abbreviations

ALI: Acute lung injury
ARIs: Acute respiratory infections
BP: Biological process
CC: Cellular component
DXMS: Dexamethasone
GM-CSF: Granulocyte macrophage colony-stimulating factor
GO: Gene ontology
HE: Hematoxylin and eosin
IFN: Interferon
IL: Interleukin
KEGG: Kyoto Encyclopedia of Genes and Genomes
LPS: Lipopolysaccharide
MF: Molecular function
OLFM4: Olfactomedin 4
QBH: Qinbaohong Zhieke oral liquid
RANTES: Regulated upon activation, normal T cell expressed and presumably secreted
RIPA: Radio radioimmunoprecipitation assay
SD: Standard deviation
TCM: Traditional Chinese medicine
TCMSP: Traditional Chinese medicine systems pharmacology
TMT: Tandem mass tag
TNF: Tumor necrosis factor.

## Supplementary Materials

Supplementary 1 The content of QBH was determined by high-performance liquid chromatography (HPLC) analysis (general rule 0512) based on Chinese Pharmacopoeia 2020. Figure 1 showed HPLC chromatograms of the standard baicalin and QBH sample. Figure 2 showed HPLC chromatograms of the standard farrerol and QBH sample.

Supplementary 2 The detailed results of network pharmacology analysis were displayed. Table S1 showed 324 chemical ingredients of QBH collected from the TCMSP database and documentary records. Table S2 showed 358 target genes of respiratory tract infections acquired from the Human Phenotype Ontology and DisGeNET databases. Table S3 showed 65 overlapping targets identified by QBH and respiratory tract infections.
