# Supplementary material for: Qinbaohong Zhike Oral Liquid Attenuates LPS-Induced Acute Lung Injury in Immature Rats by Inhibiting OLFM4
Source: Oxid Med Cell Longev. 2022 Aug 16;2022:7272371. doi: 10.1155/2022/7272371 (PMC9400428; doi:10.1155/2022/7272371)
Supplement: Supplementary 1 — The content of QBH was determined by high-performance liquid chromatography (HPLC) analysis (general rule 0512) based on Chinese Pharmacopoeia 2020. Figure 1 showed HPLC chromatograms of the standard baicalin and QBH sample. Figure 2 showed HPLC chromatograms of the standard farrerol and QBH sample. [file 7272371.f1.docx]

**Supplementary Material 1****--Content Determination of Qinbaohong Zhike**

**Oral Liquid**

The content of Qinbaohong Zhike Oral Liquid (QBH) was determined by high performance liquid chromatography (HPLC) analysis (general rule 0512) based on Chinese Pharmacopoeia 2020.

1. **Methods**
   1. **Materials**

The reference standards of baicalin (Batch No. 182307-202104, Purity≥99.8%) and farrerol (Batch No. 183415-202102, Purity≥99.8%) were purchased from National Institutes for Food and Drug Control (Beijing, China). QBH was provided by Heilongjiang Bifu Jinbeiyao Biopharmaceutical Co. Ltd., China (Batch No. 21100209, 10 mL/bottle). All solvents (methanol, trichloromethane and glacial acetic acid) were HPLC grade (Thermo Fisher, MA, USA). Water was purified by a Milli-Q system (Millipore, MA, USA).

**1.2 Determination of** [***Scutellaria baicalensis***](https://www.youdao.com/w/Scutellaria%20baicalensis/#keyfrom=E2Ctranslation)

**1.2.1 Chromatographic condition**

The chromatographic column was C18 column (Agilent Zorbax, 150 mm × 4.6 mm, 5 μm), the mobile phase was methanol-water-glacial acetic acid (45: 55: 1, v/v/v), and the detection wavelength was 274 nm.

**1.2.2 Preparation of control solution**

10.0 mg reference substance baicalin was taken precisely, and added to the 100-mL volumetric flask. It was dissolved in 50% methanol, diluted to the volume, and then mixed to get the control solution (0.10 mg/mL).

**1.2.3 Preparation of sample solution**

5 bottles of QBH were mixed, and 5 mL sample solution was taken precisely. It was transferred to a 25-mL volumetric flask, diluted with methanol to the volume, and mixed. The sample solution was left to stand for about 10 min, and filtered. The filter liquor (5.0 mL) was measured precisely, placed in a 50-mL volumetric flask, diluted with 50% methanol to the volume, and mixed. Then it was filtered to get the clear sample fluid.

**1.2.4 HPLC detection**

The control solution (10 μL) and the sample solution (10 μL) were measured by HPLC (Waters 2695, MA, USA). The number of theoretical plates calculated for baicalin peak was not lower than 1500.

**1.3 Determination of *Rhododendron dauricum* L.**

**1.3.1 Chromatographic condition**

The chromatographic column was C18 column (Agilent Zorbax, 150 mm × 4.6 mm, 5 μm), the mobile phase was methanol-water (58: 42, v/v), and the detection wavelength was 295 nm.

**1.3.2 Preparation of control solution**

10.0 mg substance farrerol was taken precisely, and added to the 1000-mL volumetric flask. It was dissolved in methanol, diluted to the volume, and then mixed to get the control solution (10 μg /mL).

**1.3.3 Preparation of sample solution**

5 bottles of QBH were mixed, and 10 mL sample solution was taken precisely. It was transferred to a 50-mL volumetric flask, diluted with methanol to the volume, and mixed. The sample solution was left to stand for about 10 min, and filtered. The filter liquor (20.0 mL) was measured precisely and dried. The residues were dissolved in 20 mL water, and trichloromethane (25mL, 25mL, 20mL, 20mL, 15mL) was added to extract 5 times with vibration. The extractions were merged and dried. The residues were dissolved in a right amount of methanol, and transferred into a 10-mL volumetric flask. The solution was diluted with methanol to the volume, shaken up and filtered to get the clear sample fluid.

**1.3.4 HPLC detection**

The control solution (10 μL) and the sample solution (10 μL) were measured by HPLC (Waters 2695, MA, USA). The number of theoretical plates calculated for farrerol peak was not lower than 5000.

1. **Results**

**2.1 Content determination of baicalin in** [***Scutellaria baicalensis***](https://www.youdao.com/w/Scutellaria%20baicalensis/#keyfrom=E2Ctranslation)

The retention times of baicalin in the control solution and the sample solution were 19.850 min and 20.316 min, respectively (Figure 1). The content determination of baicalin in the sample was 7.8 mg/mL, which had reached the standard of Chinese Pharmacopoeia 2020.

FIGURE 1: HPLC chromatograms of the standard baicalin (a)

and QBH sample (b).


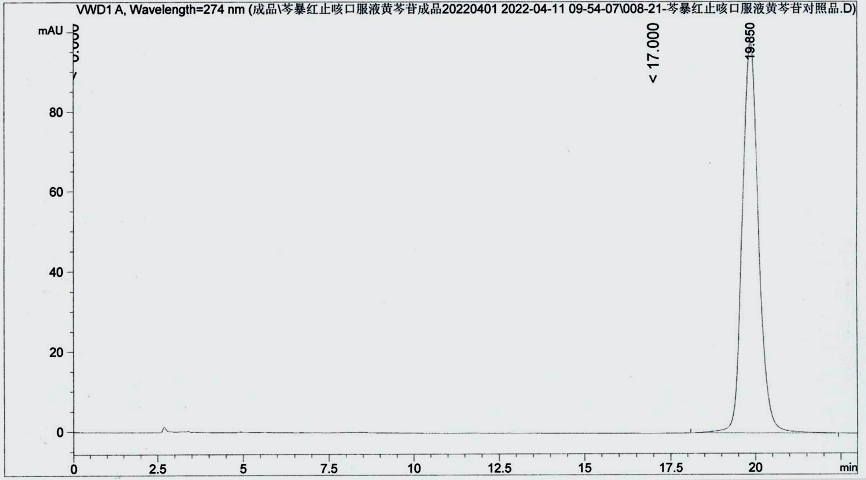


(a)

(b)


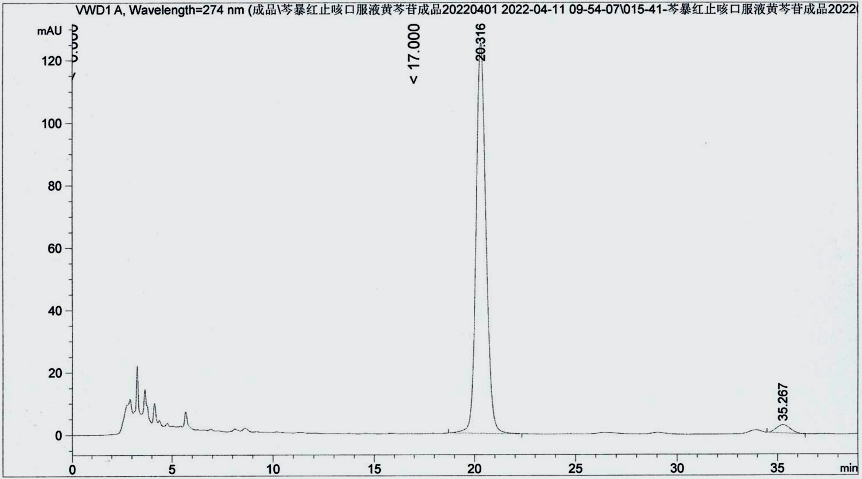


**2.2 Content determination of farrerol in *Rhododendron dauricum* L.**

The retention times of farrerol in the control solution and the sample solution were 23.347 min and 23.510 min, respectively (Figure 2). The content determination of farrerol in the sample was 43.2 μg/mL, which had reached the standard of Chinese Pharmacopoeia 2020.

FIGURE 2: HPLC chromatograms of the standard farrerol (a)

and QBH sample (b).


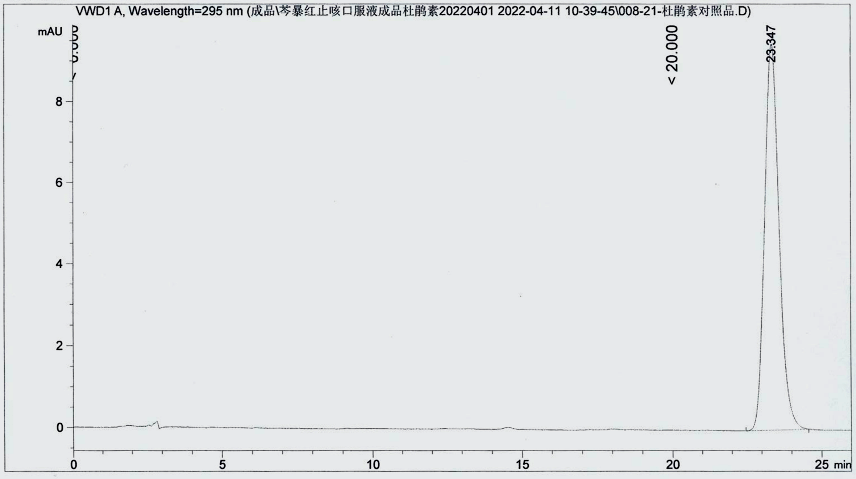


(a)


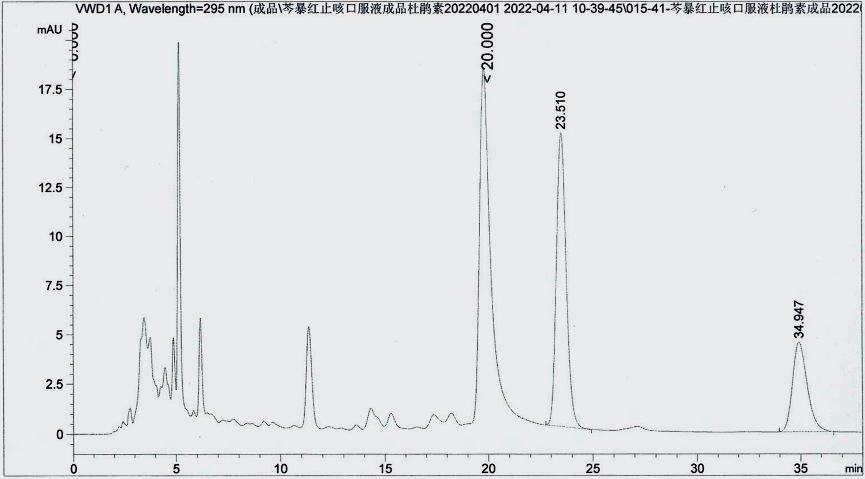


(b)
