# Supplementary material for: Qinbaohong Zhike Oral Liquid Attenuates LPS-Induced Acute Lung Injury in Immature Rats by Inhibiting OLFM4
Source: Oxid Med Cell Longev. 2022 Aug 16;2022:7272371. doi: 10.1155/2022/7272371 (PMC9400428; doi:10.1155/2022/7272371)
Supplement: Supplementary 2 — The detailed results of network pharmacology analysis were displayed. Table S1 showed 324 chemical ingredients of QBH collected from the TCMSP database and documentary records. Table S2 showed 358 target genes of respiratory tract infections acquired from the Human Phenotype Ontology and DisGeNET databases. Table S3 showed 65 overlapping targets identified by QBH and respiratory tract infections. [file 7272371.f2.docx]

**Supplementary Material 2--Network Pharmacology Result**

**Table S1 324 chemical ingredients of Qinbaohong Zhike Oral Liquid collected from the TCMSP database and documentary records.**

| **Traditional Chinese Medicine** | **Mol ID** | **Molecule Name** | **OB** | **DL** |
| --- | --- | --- | --- | --- |
| *Scutellaria baicalensis* Georgi | MOL001300 | PEL | 44.03 | 0.02 |
|  | MOL001689 | acacetin | 34.97 | 0.24 |
|  | MOL000173 | wogonin | 30.68 | 0.23 |
|  | MOL013068 | Oroxindin | 7.07 | 0.77 |
|  | MOL000018 | (+/-)-Isoborneol | 86.98 | 0.05 |
|  | MOL000219 | BOX | 31.55 | 0.02 |
|  | MOL000228 | (2R)-7-hydroxy-5-methoxy-2-phenylchroman-4-one | 55.23 | 0.2 |
|  | MOL000024 | alpha-humulene | 22.98 | 0.06 |
|  | MOL000254 | eugenol | 56.24 | 0.04 |
|  | MOL002560 | chrysin | 22.61 | 0.18 |
|  | MOL002573 | β-patchoulene | 50.69 | 0.11 |
|  | MOL002714 | baicalein | 33.52 | 0.21 |
|  | MOL002737 | scutellarein | 18.97 | 0.24 |
|  | MOL002908 | 5,8,2'-Trihydroxy-7-methoxyflavone | 37.01 | 0.27 |
|  | MOL002909 | 5,7,2,5-tetrahydroxy-8,6-dimethoxyflavone | 33.82 | 0.45 |
|  | MOL002910 | Carthamidin | 41.15 | 0.24 |
|  | MOL002911 | 2,6,2',4'-tetrahydroxy-6'-methoxychaleone | 69.04 | 0.22 |
|  | MOL002912 | Dihydrobaicalin | 20.85 | 0.75 |
|  | MOL002913 | Dihydrobaicalin_qt | 40.04 | 0.21 |
|  | MOL002915 | Salvigenin | 49.07 | 0.33 |
|  | MOL002916 | 2-(2,6-dihydroxyphenyl)-3,5,7-trihydroxy-chromone | 16.44 | 0.27 |
|  | MOL002917 | 5,2',6'-Trihydroxy-7,8-dimethoxyflavone | 45.05 | 0.33 |
|  | MOL002918 | Ganhuangenin | 1.34 | 0.37 |
|  | MOL002919 | Viscidulin III | 14.36 | 0.37 |
|  | MOL000169 | alpha-Guaiene | 25.93 | 0.07 |
|  | MOL002921 | (2S,3R,4R,5R,6S)-2-[(2R,3R,4S,5R,6R)-3,5-dihydroxy-2-[2-(3-hydroxy-4-methoxy-phenyl)ethoxy]-6-methylol-tetrahydropyran-4-yl]oxy-6-methyl-tetrahydropyran-3,4,5-triol | 12.69 | 0.67 |
|  | MOL002922 | 5-(2-hydroxyethyl)-2-methoxyphenol | 31.95 | 0.04 |
|  | MOL002923 | darendoside B | 10.75 | 0.59 |
|  | MOL002924 | darendoside B_qt | 10.05 | 0.22 |
|  | MOL002925 | 5,7,2',6'-Tetrahydroxyflavone | 37.01 | 0.24 |
|  | MOL002926 | dihydrooroxylin A | 38.72 | 0.23 |
|  | MOL002927 | Skullcapflavone II | 69.51 | 0.44 |
|  | MOL002928 | oroxylin a | 41.37 | 0.23 |
|  | MOL002931 | scutellarin | 2.64 | 0.79 |
|  | MOL002932 | Panicolin | 76.26 | 0.29 |
|  | MOL002933 | 5,7,4'-Trihydroxy-8-methoxyflavone | 36.56 | 0.27 |
|  | MOL002934 | NEOBAICALEIN | 104.34 | 0.44 |
|  | MOL002935 | Baicalin | 29.53 | 0.77 |
|  | MOL002936 | 5,8-Dihydroxy-6,7-dimethoxyflavone | 5.74 | 0.29 |
|  | MOL002937 | DIHYDROOROXYLIN | 66.06 | 0.23 |
|  | MOL000357 | Sitogluside | 20.63 | 0.62 |
|  | MOL000359 | sitosterol | 36.91 | 0.75 |
|  | MOL000396 | (+)-Syringaresinol | 3.29 | 0.72 |
|  | MOL000458 | campesterol | 5.57 | 0.72 |
|  | MOL000525 | Norwogonin | 39.4 | 0.21 |
|  | MOL000552 | 5,2'-Dihydroxy-6,7,8-trimethoxyflavone | 31.71 | 0.35 |
|  | MOL000612 | (-)-alpha-cedrene | 55.56 | 0.1 |
|  | MOL000007 | Cosmetin | 9.68 | 0.74 |
|  | MOL000709 | (S)-Matsutake alcohol | 40.11 | 0.01 |
|  | MOL000715 | l-Menthone | 57.9 | 0.03 |
|  | MOL000008 | apigenin | 23.06 | 0.21 |
|  | MOL003127 | Germacrene D | 19.22 | 0.06 |
|  | MOL000073 | ent-Epicatechin | 48.96 | 0.24 |
|  | MOL000131 | EIC | 41.9 | 0.14 |
|  | MOL000303 | caprylic acid | 16.4 | 0.02 |
|  | MOL000449 | Stigmasterol | 43.83 | 0.76 |
|  | MOL000610 | TRD | 17.89 | 0.03 |
|  | MOL000654 | Methyl montanate | 13.2 | 0.48 |
|  | MOL000669 | (S)-camphor | 21.68 | 0.05 |
|  | MOL000676 | DBP | 64.54 | 0.13 |
|  | MOL000714 | Hyacinthin | 38.65 | 0.02 |
|  | MOL000717 | d-isomenthone | 61.2 | 0.03 |
|  | MOL000771 | p-coumaric acid | 43.29 | 0.04 |
|  | MOL000789 | jatrorrizine | 19.65 | 0.59 |
|  | MOL000867 | Heptadekan | 8.64 | 0.07 |
|  | MOL000868 | LFA | 8.46 | 0.13 |
|  | MOL000869 | Henicosane | 8.41 | 0.15 |
|  | MOL000870 | HEXATRIACONTANE | 7.95 | 0.41 |
|  | MOL000879 | methyl palmitate | 18.09 | 0.12 |
|  | MOL000885 | Dodekan | 17.74 | 0.02 |
|  | MOL001132 | longipinene | 17.01 | 0.12 |
|  | MOL001386 | Methyl laurate | 21.75 | 0.05 |
|  | MOL001392 | Methyl myristate | 19.68 | 0.08 |
|  | MOL001458 | coptisine | 30.67 | 0.86 |
|  | MOL001490 | bis[(2S)-2-ethylhexyl] benzene-1,2-dicarboxylate | 43.59 | 0.35 |
|  | MOL001506 | Supraene | 33.55 | 0.42 |
|  | MOL001578 | Hypnon | 48.19 | 0.02 |
|  | MOL001817 | Methyl stearate | 16.8 | 0.16 |
|  | MOL001818 | Methyl palmitelaidate | 34.61 | 0.12 |
|  | MOL001889 | Methyl linolelaidate | 41.93 | 0.17 |
|  | MOL001972 | Pulegone | 51.6 | 0.03 |
|  | MOL002027 | Methyl behenate | 14.96 | 0.29 |
|  | MOL002046 | hexanoic acid | 73.08 | 0.01 |
|  | MOL002137 | OCT | 29.72 | 0.01 |
|  | MOL002202 | Tetramethyl pyrazine | 20.01 | 0.03 |
|  | MOL002378 | UND | 17.15 | 0.02 |
|  | MOL002819 | catalpol | 5.07 | 0.44 |
|  | MOL002879 | Diop | 43.59 | 0.39 |
|  | MOL002897 | epiberberine | 43.09 | 0.78 |
|  | MOL003050 | nonanoic acid | 40.51 | 0.02 |
|  | MOL003055 | heptadecyloxirane | 12.44 | 0.15 |
|  | MOL003393 | (1S,4S)-7-isopropylidene-1,4-dimethyl-2,3,4,5,6,8-hexahydro-1H-azulene | 24.38 | 0.07 |
|  | MOL003475 | 9-Cedranone | 67.6 | 0.12 |
|  | MOL003535 | 1,1,6-trimethyl-2H-naphthalene | 24.94 | 0.06 |
|  | MOL003568 | Patchoulene | 49.06 | 0.11 |
|  | MOL003920 | Methyl icosanoate | 15.79 | 0.22 |
|  | MOL004464 | MEHQ | 43.98 | 0.02 |
|  | MOL004682 | Methyl octylate | 18.71 | 0.02 |
|  | MOL004684 | methyl (E)-octadec-2-enoate | 29.84 | 0.17 |
|  | MOL005021 | Mipax | 57.4 | 0.06 |
|  | MOL005224 | TETRATETRACONTANE | 7.82 | 0.25 |
|  | MOL005272 | 13-Tetradecenyl acetate | 36.76 | 0.1 |
|  | MOL005368 | Methyl tricosanoate | 14.61 | 0.33 |
|  | MOL005402 | Methyl margarate | 17.41 | 0.14 |
|  | MOL005577 | undecanal | 22.9 | 0.03 |
|  | MOL005841 | TBP | 27.76 | 0.06 |
|  | MOL006219 | Clorius | 45.99 | 0.02 |
|  | MOL006312 | Azulol | 15.15 | 0.07 |
|  | MOL006370 | 5-o-caffeoylquinic acid | 19.61 | 0.33 |
|  | MOL007197 | DFA | 31.13 | 0.05 |
|  | MOL007792 | Isomartynoside | 13.98 | 0.56 |
|  | MOL008151 | METHYL NONADECANOATE | 16.27 | 0.19 |
|  | MOL008206 | Moslosooflavone | 44.09 | 0.25 |
|  | MOL008595 | methyl henicosanoate | 15.36 | 0.26 |
|  | MOL008615 | Methyl 9-oxononanoate | 24.02 | 0.04 |
|  | MOL009520 | 3,8-dimethylundecane | 4.72 | 0.03 |
|  | MOL009730 | methyl icos-11-enoate | 29.49 | 0.23 |
|  | MOL009734 | Methyl lignocerate | 14.27 | 0.37 |
|  | MOL010206 | Methyl isoheptadecanoate | 21.72 | 0.14 |
|  | MOL010415 | 11,13-Eicosadienoic acid, methyl ester | 39.28 | 0.23 |
|  | MOL010563 | Methyl (Z)-cinnamate | 37.2 | 0.04 |
|  | MOL011081 | Linolenic acid methyl ester | 46.15 | 0.17 |
|  | MOL011322 | Diisobutyl succinate | 39.54 | 0.06 |
|  | MOL012240 | 2',3',5,7-tetrahydroxyflavone | 25.75 | 0.24 |
|  | MOL012245 | 5,7,4'-trihydroxy-6-methoxyflavanone | 36.63 | 0.27 |
|  | MOL012246 | 5,7,4'-trihydroxy-8-methoxyflavanone | 74.24 | 0.26 |
|  | MOL012266 | rivularin | 37.94 | 0.37 |
|  | MOL012267 | Scutevulin | 20.67 | 0.27 |
|  | MOL012564 | 3,7-dimethylnonane | 15.31 | 0.02 |
|  | MOL013062 | BZQ | 58.62 | 0.06 |
|  | MOL013161 | METHYL HEXACOSANOATE | 13.68 | 0.43 |
| *Rhododendron dauricum* L. | MOL010259 | Apple oil | 22.26 | 0.03 |
|  | MOL000103 | PHB | 30.15 | 0.03 |
|  | MOL000105 | protocatechuic acid | 25.37 | 0.04 |
|  | MOL011001 | Mesityloxid | 50.93 | 0.01 |
|  | MOL001123 | muurolene | 19.5 | 0.08 |
|  | MOL000114 | vanillic acid | 35.47 | 0.04 |
|  | MOL000116 | Nonanal | 40.28 | 0.02 |
|  | MOL001168 | (1S,2S)-2-isopropenyl-4-isopropylidene-1-methyl-1-vinylcyclohexane | 34.47 | 0.06 |
|  | MOL001189 | cis-caryophyllene | 29.71 | 0.09 |
|  | MOL011969 | Demethylfuropinnarin | 41.31 | 0.21 |
|  | MOL001201 | (1R,5R,7S)-4,7-dimethyl-7-(4-methylpent-3-enyl)bicyclo[3.1.1]hept-3-ene | 16.23 | 0.09 |
|  | MOL012265 | ARC | 20.61 | 0.13 |
|  | MOL012409 | (2R-cis)-1,2,3,4,4a,5,6,7-Octahydro-alpha,alpha,4a,8-tetramethyl-2-naphthalenemethanol | 24.28 | 0.1 |
|  | MOL012410 | (1R,3E,7E,11R)-1,5,5,8-tetramethyl-12-oxabicyclo[9.1.0]dodeca-3,7-diene | 33.23 | 0.1 |
|  | MOL012411 | 1,2,3,6-tetramethyldicyclo[2.2.2]oct-2-ene | 50.48 | 0.06 |
|  | MOL012412 | 1,5,5,8-tetramethyl-3,7-cycloundecadien-1-ol | 33.92 | 0.07 |
|  | MOL012413 | 1,5-dimethyl-6-methylethenylspiro[2.4]heptane | 40.69 | 0.06 |
|  | MOL012414 | 10,10-Dimethyl-2,6-dimethylene bicyclo[7.2.0]undecan-5beta-ol | 35.27 | 0.1 |
|  | MOL012415 | 1-cis,5-trans Germacrone | 29.62 | 0.07 |
|  | MOL012416 | 2,3,4-Trimethoxyacetophenone | 19.1 | 0.06 |
|  | MOL012417 | Picoline | 47.89 | 0.01 |
|  | MOL012418 | Amyl cyclopentenone | 124.71 | 0.03 |
|  | MOL012419 | 4a,8-dimethyl-2-(1-methylethenylethyl)-1,2,3,4,4a,5,6,8a-octahydronaphthalene | 16.37 | 0.11 |
|  | MOL012420 | 4a-methyl-1-methylethenyl-7-(1-methylethenylethyl) decahydronaphthalene | 14.73 | 0.14 |
|  | MOL012421 | 5-methyl kaempferol | 41.4 | 0.24 |
|  | MOL012422 | 2-Ethyl-5-methylpyridine | 48.65 | 0.02 |
|  | MOL012423 | 5-methyl-2-isopropyl-9-methylethenyldicyclo[4.4.0]dec-1-ene | 19.69 | 0.1 |
|  | MOL012424 | 6-Tetradecanone | 9.65 | 0.05 |
|  | MOL012425 | 7,11-dimethyl-3-methylethenyl-1,6,10-dodecatriene | 3.49 | 0.07 |
|  | MOL012426 | 7-methyl-4-methylethenyl-1,2,3,4,4a,5,6,8a-octahydronaphthalene | 46.93 | 0.07 |
|  | MOL012427 | 8-demethyl farrerol | 46.28 | 0.24 |
|  | MOL012428 | Weedout | 19.14 | 0.13 |
|  | MOL012429 | Cyclohexadecane | 16.61 | 0.09 |
|  | MOL012430 | eriodictin | 29.48 | 0.74 |
|  | MOL012431 | (2E,4E,6E)-3,7,11-trimethyldodeca-2,4,6,10-tetraene | 17.59 | 0.05 |
|  | MOL012432 | Farrerol | 42.65 | 0.26 |
|  | MOL012433 | Fastigilin B | 109.45 | 0.4 |
|  | MOL012434 | Germanicol | 11 | 0.76 |
|  | MOL012435 | Gossypetin-3-beta-D-(2-O-beta-D-glucopyranosidoglucopyranoside)-8-beta-D-glucopyranoside | 3.01 | 0.31 |
|  | MOL012436 | Grayanotoxin I | 60.55 | 0.61 |
|  | MOL012437 | graynotoxin Ⅱ | 47.57 | 0.45 |
|  | MOL012438 | Hexyl isovalerate | 18.84 | 0.03 |
|  | MOL012439 | (1Z,4E,8E)-2,6,6,9-tetramethylcycloundeca-1,4,8-triene | 22.83 | 0.06 |
|  | MOL012440 | Isohyperoside acetate | 1.87 | 0.77 |
|  | MOL012441 | (1S,4aS,8aS)-7-isopropylidene-1,4a-dimethyl-1-decalinol | 33.39 | 0.1 |
|  | MOL012442 | kaur-16-ene | 15.93 | 0.27 |
|  | MOL012443 | Ambrettolid | 40.59 | 0.14 |
|  | MOL012444 | poriol | 38.45 | 0.24 |
|  | MOL012445 | 4-[(3R)-3-hydroxybutyl]phenol | 43.89 | 0.04 |
|  | MOL012446 | Rhodojaponin IV | 17.61 | 0.72 |
|  | MOL012447 | santolina triene | 36.56 | 0.02 |
|  | MOL012448 | Syrionylglycerol-beta-syringaresinol_qt | 8.4 | 0.6 |
|  | MOL012449 | β-lonene | 45.93 | 0.06 |
|  | MOL012450 | γ-selinene | 18.02 | 0.08 |
|  | MOL000125 | (-)-alpha-Pinene | 46.25 | 0.05 |
|  | MOL000126 | (-)-nopinene | 44.84 | 0.05 |
|  | MOL000130 | CAM | 67.17 | 0.05 |
|  | MOL001335 | WLN: Q1R | 58.68 | 0.01 |
|  | MOL001388 | (+)-Ledol | 16.96 | 0.12 |
|  | MOL000511 | ursolic acid | 16.77 | 0.75 |
|  | MOL001556 | Isocaryophyllene | 27.3 | 0.09 |
|  | MOL001570 | 1,4,7,-Cycloundecatriene, 1,5,9,9-tetramethyl-, Z,Z,Z- | 20.82 | 0.06 |
|  | MOL001606 | BB_NC-0668 | 35.57 | 0.08 |
|  | MOL001641 | METHYL LINOLEATE | 41.93 | 0.17 |
|  | MOL001719 | 2-[(2S,5R)-5-ethenyl-5-methyloxolan-2-yl]propan-2-ol | 68.08 | 0.04 |
|  | MOL000172 | Furol | 34.35 | 0.01 |
|  | MOL001736 | (-)-taxifolin | 60.51 | 0.27 |
|  | MOL001807 | Cedar acid | 47.78 | 0.06 |
|  | MOL001862 | Cadalin | 12.96 | 0.08 |
|  | MOL001942 | isoimperatorin | 45.46 | 0.23 |
|  | MOL000196 | L-Bornyl acetate | 65.52 | 0.08 |
|  | MOL000197 | Myrcene | 24.96 | 0.02 |
|  | MOL002002 | cis-Carveol | 45.61 | 0.03 |
|  | MOL002003 | (-)-Caryophyllene oxide | 32.67 | 0.13 |
|  | MOL002006 | Isocembrol | 15.06 | 0.17 |
|  | MOL002008 | myricetin | 13.75 | 0.31 |
|  | MOL000201 | p-Ocimene | 15.06 | 0.02 |
|  | MOL000202 | Moslene | 33.02 | 0.02 |
|  | MOL000207 | Methyleugenol | 73.36 | 0.04 |
|  | MOL002085 | alpha-Cubebene | 16.73 | 0.11 |
|  | MOL002112 | alpha-Selinene | 31.81 | 0.1 |
|  | MOL002132 | (2R,4aR)-2-isopropenyl-4a,8-dimethyl-2,3,4,5,6,7-hexahydro-1H-naphthalene | 22.13 | 0.08 |
|  | MOL002190 | Cedrene | 51.14 | 0.11 |
|  | MOL002215 | Oleanic acid | 8.41 | 0.77 |
|  | MOL000023 | Hemo-sol | 39.84 | 0.02 |
|  | MOL000232 | ()-alpha-Terpineol | 46.3 | 0.03 |
|  | MOL002351 | 3-Hexenol | 62.74 | 0.01 |
|  | MOL002453 | (-)-Comphene | 34.98 | 0.04 |
|  | MOL002458 | ZINC01850974 | 78.86 | 0.05 |
|  | MOL002502 | copaene | 24.08 | 0.12 |
|  | MOL002534 | 1,6-dimethyl-4-isopropyl-1,2,3,4,4a,7-hexahydronaphthalene | 17.14 | 0.08 |
|  | MOL002538 | Benzylacetone | 25.78 | 0.03 |
|  | MOL002558 | Skimmetin | 27.37 | 0.05 |
|  | MOL002675 | Hexenal | 46.01 | 0.01 |
|  | MOL000268 | (1S,5S)-1-isopropyl-4-methylenebicyclo [3.1.0] hexane | 46.21 | 0.04 |
|  | MOL002818 | Piceol | 36.8 | 0.03 |
|  | MOL002835 | δ-cadinol | 17.13 | 0.08 |
|  | MOL003028 | Eudesmol | 35.38 | 0.09 |
|  | MOL000305 | lauric acid | 23.59 | 0.04 |
|  | MOL000032 | beta-Eudesmol | 26.09 | 0.1 |
|  | MOL003484 | PEY | 25.7 | 0.1 |
|  | MOL003493 | naphthalene | 27.55 | 0.03 |
|  | MOL003571 | spathulenol | 81.61 | 0.12 |
|  | MOL000040 | Scopoletol | 27.77 | 0.08 |
|  | MOL004020 | gossypetin | 35 | 0.31 |
|  | MOL004079 | (E)-calamenene | 17.31 | 0.08 |
|  | MOL004093 | Azaleatin | 54.28 | 0.3 |
|  | MOL004107 | Syrionylglycerol-beta-syringaresinol | 3.01 | 0.09 |
|  | MOL004134 | Orcin | 48.14 | 0.02 |
|  | MOL004368 | Hyperin | 6.94 | 0.77 |
|  | MOL004713 | alpha-Eudesmol | 25.02 | 0.1 |
|  | MOL005125 | ANN | 29.69 | 0.03 |
|  | MOL000513 | 3,4,5-trihydroxybenzoic acid | 31.69 | 0.04 |
|  | MOL005136 | Andromedotoxin | 55.97 | 0.61 |
|  | MOL005141 | graynotoxin Ⅲ | 47.01 | 0.48 |
|  | MOL005270 | n-Heptadecanol | 12.97 | 0.09 |
|  | MOL005501 | Green Oil | 17.74 | 0.1 |
|  | MOL000579 | hydroquinone | 29.26 | 0.02 |
|  | MOL005964 | β-panasinsene | 56.28 | 0.12 |
|  | MOL000609 | (1R,5S)-7,7-dimethyl-4-bicyclo [3.1.1] hept-3-enecarboxaldehyde | 40.64 | 0.06 |
|  | MOL000615 | delta-amorphene | 17.95 | 0.08 |
|  | MOL006289 | cis-Z-alpha-Bisabolene epoxide | 21.33 | 0.09 |
|  | MOL000668 | PENTYLFURAN | 54.59 | 0.02 |
|  | MOL006702 | (1R,3S,6S)-3,7,7-trimethylbicyclo [4.1.0] hept-4-ene | 41.1 | 0.04 |
|  | MOL000671 | ()-Menthol | 59.33 | 0.03 |
|  | MOL000712 | o-Cymol | 51.89 | 0.02 |
|  | MOL000724 | Geranylacetone | 18.66 | 0.04 |
|  | MOL007330 | MENTHOL | 43.31 | 0.03 |
|  | MOL007979 | Avicularin | 2.06 | 0.7 |
|  | MOL000861 | Healip | 11.65 | 0.22 |
|  | MOL000873 | CYH | 74.99 | 0.01 |
|  | MOL000876 | (6R,10R)-6,10,14-trimethylpentadecan-2-one | 23.3 | 0.1 |
|  | MOL000878 | Farnesylacetone | 37.84 | 0.1 |
|  | MOL000886 | tetradecane | 15.94 | 0.04 |
|  | MOL000890 | (+)-alpha-Curcumene | 26.56 | 0.06 |
|  | MOL000910 | Germacron | 32.5 | 0.07 |
|  | MOL000911 | Terpilene | 33.95 | 0.02 |
|  | MOL000922 | (R)-p-Menth-1-en-4-ol | 32.16 | 0.03 |
|  | MOL000923 | ACETIC ACID,BORNYL ESTER | 67.15 | 0.08 |
|  | MOL000927 | α-muurolene | 17.24 | 0.08 |
|  | MOL009451 | .alpha.-Carene | 48.01 | 0.04 |
|  | MOL009782 | Dihydroresveratrol | 87.27 | 0.11 |
|  | MOL000098 | quercetin | 46.43 | 0.28 |
| *Syringa reticulata* (Blume) H. Hara | - | oleoside 11-methyl ester | - | - |
|  | MOL005176 | oleuropein | 4.95 | 0.72 |
|  | - | ligstroside | - | - |
|  | - | jaspolyoside | - | - |
|  | - | reticuloside | - | - |
|  | - | 2″-epifraxamoside | - | - |
|  | - | 8-[2- (3, 4-dihydroxy-phenyl) -ethoxycarbonymethyl]-carboxylic acid methyl ester | - | - |
|  | MOL000347 | syringin | 14.64 | 0.32 |
|  | - | sinapylaldehyde 4-O-β-D-glucopyranoside | - | - |
|  | - | coniferyaldehyde glucoside | - | - |
|  | - | isosyringinoside | - | - |
|  | MOL003837 | esculetin | 22.97 | 0.07 |
|  | MOL003177 | syringaldehyde | 67.06 | 0.05 |
|  | MOL002049 | coniferyl aldehyde | 49.26 | 0.05 |
|  | MOL000211 | betulinic acid | 55.38 | 0.78 |
|  | MOL005173 | oleoside dimethyl ester | 55.9 | 0.1 |
|  | - | beta- sitosterol | - | - |
|  | - | n-triaconatanoic acid | - | - |
|  | - | 2- (4-hydroxyphenyl) -ethyl-1-dodecyloctadecanoate | - | - |
|  | - | pinoresinol-4-O-β-monoglycoside | - | - |
|  | - | syringaresinol-4-O-bis-β-D-monoglucoside | - | - |
|  | - | syringaresinol-4,4"-O-bis-β-D-glucoside | - | - |
|  | - | cyclo-olivil6-O-β-D-glucoside | - | - |
|  | - | olivil4-O-β-D-glucopyranoside | - | - |
|  | - | olivil4"-O-β-D-glucopyranoside | - | - |
|  | - | armandiside | - | - |
|  | - | 3,4-dihydroxyphenyl | - | - |
|  | - | 2-(3,4-dihydroxy)-phenyl-ethyl-β-D-glucopyranoside | - | - |
|  | - | Sakuranetin | - | - |
|  | - | Sakuranetin-5-O-β-D-xylopyranoside | - | - |
|  | - | quercetin-3-O-β-D-glucopyranoside | - | - |
|  | MOL006775 | epiafzelechin | 23.74 | 0.21 |
|  | MOL001996 | Betulonic acid | 16.83 | 0.78 |
|  | - | 9, 12-linoleic acid | - | - |
|  | - | 9, 17-octadecadienal | - | - |
|  | MOL000610 | Tridecane | 17.89 | 0.03 |
|  | MOL000003 | D-mannitol | 17.73 | 0.03 |
| *Scutellaria baicalensis* Georgi, *Rhododendron dauricum* L. | MOL000122 | 1,8-cineole | 39.73 | 0.05 |
|  | MOL000198 | (R)-linalool | 39.8 | 0.02 |
|  | MOL002914 | Eriodyctiol (flavanone) | 41.35 | 0.24 |
|  | MOL000358 | beta-sitosterol | 36.91 | 0.75 |
|  | MOL000035 | beta-Selinene | 24.39 | 0.08 |
|  | MOL000708 | WLN: VHR | 32.63 | 0.01 |
|  | MOL000860 | stearic acid | 17.83 | 0.14 |
|  | MOL000864 | MYS | 13.98 | 0.05 |
|  | MOL001393 | myristic acid | 21.18 | 0.07 |
| *Scutellaria baicalensis* Georgi, *Syringa reticulata* (Blume) H. Hara | MOL002929 | salidroside | 7.01 | 0.2 |
|  | MOL002930 | Tyrosol | 33.81 | 0.02 |
| *Rhododendron dauricum* L., *Syringa reticulata* (Blume) H. Hara | MOL000422 | kaempferol | 41.88 | 0.24 |
| *Scutellaria baicalensis* Georgi, *Rhododendron dauricum* L., *Syringa reticulata* (Blume) H. Hara | MOL000069 | palmitic acid | 19.3 | 0.1 |

**Table S2 358 target genes of respiratory tract infections acquired from the Human Phenotype Ontology and DisGeNET databases.**

| FBLN5 | PIK3CB | FOXJ1 | IGFALS | MYSM1 | COL5A2 |
| --- | --- | --- | --- | --- | --- |
| LEPR | PIK3CD | FCGR2B | IFNB1 | NBN | CREBBP |
| RPGR | PIK3CG | FCGR2A | IFNA13 | NCF1 | DNAAF1 |
| ALG12 | SELL | CYP27B1 | IFNA2 | NCF2 | DNAAF11 |
| PYROXD1 | PIK3R1 | CYP21A1P | IFNA1 | NDUFC2 | DNAAF2 |
| GSN | PLAU | ADRB2 | IDS | NFKB2 | DNAAF4 |
| TPP2 | PLG | ERVK-24 | LAMP3 | ODAD1 | DNAAF5 |
| MBL2 | MBL3P | ERVK-25 | ADA | ORC6 | DNAAF6 |
| IKBKB | SMOX | ERVK-18 | AICDA | P4HTM | DNAI2 |
| SLC12A6 | TAS2R38 | ERVK-21 | ALB | PEPD | DNAJB13 |
| PEX13 | SMN2 | ERVK-9 | ALMS1 | PGM3 | DOCK8 |
| CRP | ZAP70 | ERVK-10 | AP3B1 | PKHD1 | EP300 |
| COPD | VEGFA | MICA | BLNK | PKP1 | FCGR3A |
| IL10 | SOD1 | DEFB4B | BTK | PLOD1 | FOXN1 |
| CFTR | SSTR2 | KLRG1 | CARMIL2 | PNP | FOXP1 |
| VDR | STAT2 | IFITM3 | CD247 | POLA1 | GALNS |
| IL6 | STAT3 | CTLA4 | CD27 | PRKCD | GAS2L2 |
| FCN2 | SULT2A1 | CSF3 | CD3D | PRKDC | GLB1 |
| SARS2 | TP53 | CSF2 | CD3E | PURA | GNS |
| SARS1 | C4B | COMT | CD40LG | RAC1 | GUSB |
| IL4 | NKX2-1 | CMPK2 | CD79B | RAC2 | HGSNAT |
| STAT1 | TLR3 | TREX1 | CFAP410 | RAG1 | HPS6 |
| IL22 | TLR4 | ERVK-19 | CIITA | RAG2 | HYDIN |
| TNF | TLR5 | SLC9A6 | COL11A2 | RASGRP1 | IL17RA |
| AMBP | TNFAIP6 | VIM2P | COL13A1 | RFX5 | IL21R |
| SFTPA1 | SMN1 | GJB2 | COL5A1 | RFXANK | IL6R |
| BPIFA1 | CXCR4 | NGF | COL6A1 | RFXAP | IVNS1ABP |
| IL1B | HAMP | LCN2 | COL6A2 | RNF168 | KATNIP |
| MECP2 | PLAAT4 | CCL4L1 | COL6A3 | RNU4ATAC | MAGT1 |
| IL17A | RNASE2 | INSRR | COLQ | SCNN1A | MCIDAS |
| IFNG | TRBV20OR9-2 | IL18 | CR2 | SCNN1B | MDM4 |
| SFTPA2 | ERVK-8 | IL13 | CTCF | SCNN1G | MGP |
| CD14 | FCN3 | IL11 | CYBA | SETBP1 | MS4A1 |
| KLHL2 | SCT | IL7R | CYBB | SLC35C1 | NAGLU |
| DECR1 | SRL | IL5 | CYBC1 | SMARCD2 | NCF4 |
| DEFB1 | CCL4 | LGALS3 | DCLRE1C | SREBF1 | NEK10 |
| MFN2 | SDC1 | LSAMP | DDR2 | TBCD | NFKB1 |
| CD8A | MKKS | NFE2L2 | DNAH11 | TGFB1 | NME8 |
| DARS2 | PNPLA3 | MUC5AC | DZIP1L | TK2 | ODAD2 |
| ZBED1 | ROBO3 | ERVK-7 | EFEMP2 | TNFRSF11A | OFD1 |
| MNS1 | SFTPD | MMP9 | EGFR | TNFRSF13B | PSMB8 |
| ARHGEF1 | SLC5A2 | MMP8 | EXTL3 | TNFRSF13C | PTPN22 |
| BATF3 | TNXA | MIF | FMO3 | UNG | RSPH1 |
| TASP1 | SERPINE1 | MICB | GAS8 | USB1 | RSPH4A |
| PROS1 | NUPR1 | IGHA1 | GLI3 | USP9X | SGSH |
| FCGR2C | ESR1 | IL2RG | GNPTAB | VPS33A | SH2D1A |
| PRPS1 | PAOX | IL2 | IGHM | WAS | SH3KBP1 |
| NR1I2 | DPP4 | IL1RN | IGLL1 | WDR19 | SLC29A3 |
| CD248 | DNAH5 | HSPA1B | JAK3 | ACP5 | SPAG1 |
| TSLP | SARDH | HSPA1A | KCNJ6 | ADA2 | STXBP2 |
| PTAFR | DEFB4A | HLA-A | KPTN | ADNP | TAP1 |
| WDR1 | DEFA3 | ICOS | LAMA2 | ARSB | TAP2 |
| MKS1 | DEFA1 | GSTP1 | LAMB2 | ASAH1 | TAPBP |
| CD19 | FABP5 | NR3C1 | LEP | ATM | TBC1D24 |
| CMPK1 | FBN2 | GOT2 | LMNB1 | CCDC65 | TNFRSF1A |
| CCL4L2 | ARIH1 | GLRX | LRBA | CD79A | UNC119 |
| PF4 | FUT2 | IDH1 | LRRC56 | CD81 | XIAP |
| SERPINA1 | MTOR | IDH2 | MASP2 | CFAP298 | ZMYND10 |
| PIK3CA | DDX58 | IL1A | MED25 | CFI |  |
| NCR1 | FPR2 | IGHG3 | MTHFD1 | COG4 |  |

**Table S3 65 overlapping targets identified by Qinbaohong Zhike Oral Liquid and respiratory tract infections.**

| **Name** | **Betweenness Centrality** | **Closeness Centrality** | **Degree** |
| --- | --- | --- | --- |
| TP53 | 0.13487848 | 0.72093023 | 42 |
| IL6 | 0.086535 | 0.72941176 | 41 |
| TNF | 0.06105874 | 0.71264368 | 39 |
| EGFR | 0.07204868 | 0.68131868 | 37 |
| IL4 | 0.04455126 | 0.66666667 | 36 |
| IL10 | 0.02392443 | 0.62626263 | 35 |
| TLR4 | 0.02995151 | 0.65263158 | 34 |
| IL2 | 0.02745533 | 0.62 | 32 |
| IL1B | 0.02679406 | 0.64583333 | 32 |
| IFNG | 0.02897154 | 0.63265306 | 29 |
| CSF2 | 0.02054484 | 0.59047619 | 29 |
| LEP | 0.0231081 | 0.59615385 | 27 |
| CRP | 0.01475033 | 0.58490566 | 26 |
| IL13 | 0.01767179 | 0.57407407 | 25 |
| ESR1 | 0.01965579 | 0.56363636 | 25 |
| IL17A | 0.00579523 | 0.56880734 | 25 |
| MTOR | 0.01172154 | 0.56363636 | 24 |
| NFKB1 | 0.00691809 | 0.56363636 | 24 |
| TGFB1 | 0.00283121 | 0.55357143 | 22 |
| NR3C1 | 0.02288393 | 0.57407407 | 21 |
| JAK3 | 0.00510044 | 0.53913043 | 20 |
| TLR3 | 0.00521547 | 0.53913043 | 20 |
| PIK3R1 | 0.00469252 | 0.53913043 | 19 |
| PIK3CA | 0.00456941 | 0.53448276 | 18 |
| RAG1 | 0.00200572 | 0.52542373 | 16 |
| MIF | 8.74E-04 | 0.52542373 | 16 |
| VDR | 0.00143826 | 0.5210084 | 15 |
| IL1RN | 7.98E-05 | 0.50819672 | 15 |
| CFTR | 0.12432975 | 0.54385965 | 14 |
| ATM | 0.00433754 | 0.51239669 | 14 |
| LGALS3 | 0.00184904 | 0.51239669 | 14 |
| ADRB2 | 0.06545234 | 0.52991453 | 13 |
| PRKCD | 0.01072372 | 0.5210084 | 13 |
| IKBKB | 0.00181805 | 0.50819672 | 13 |
| RAG2 | 0.00113254 | 0.50819672 | 13 |
| PLG | 4.78E-04 | 0.51239669 | 13 |
| IL17RA | 0 | 0.49206349 | 12 |
| SREBF1 | 0.00158352 | 0.5 | 11 |
| PRKDC | 0.00171754 | 0.50406504 | 11 |
| PIK3CD | 7.83E-04 | 0.48062016 | 11 |
| TNFRSF13C | 1.71E-04 | 0.50406504 | 11 |
| PF4 | 4.41E-05 | 0.48062016 | 10 |
| PIK3CB | 5.69E-04 | 0.47692308 | 10 |
| SOD1 | 0.00182763 | 0.48818898 | 10 |
| PIK3CG | 8.56E-04 | 0.47692308 | 9 |
| SULT2A1 | 0.00201841 | 0.48062016 | 8 |
| ADA | 0.0638684 | 0.484375 | 7 |
| NKX2-1 | 8.16E-04 | 0.47328244 | 7 |
| CYP27B1 | 6.49E-04 | 0.47328244 | 6 |
| NR1I2 | 0.00112328 | 0.47328244 | 6 |
| COMT | 5.95E-04 | 0.43971631 | 5 |
| GLI3 | 4.18E-04 | 0.45255474 | 5 |
| SCNN1A | 0.00213879 | 0.37349398 | 4 |
| RNASE2 | 2.60E-04 | 0.42758621 | 4 |
| PNP | 0.03563306 | 0.33879781 | 3 |
| SCNN1B | 0 | 0.3583815 | 3 |
| SCNN1G | 0 | 0.3583815 | 3 |
| SCT | 0 | 0.36904762 | 2 |
| PRPS1 | 0.00141388 | 0.31632653 | 2 |
| POLA1 | 0.02818166 | 0.43971631 | 2 |
| KCNJ6 | 0 | 0.34831461 | 1 |
| SLC9A6 | 0 | 0.35428571 | 1 |
| CMPK2 | 0 | 0.25409836 | 1 |
| COLQ | 0 | 1 | 1 |
| PLOD1 | 0 | 1 | 1 |
